# Two new drimane sesquiterpenoids from cultures of the basidiomycete *Trichaptum biforme*

**DOI:** 10.1007/s13659-013-0030-y

**Published:** 2013-07-11

**Authors:** Xiao-Yan Yang, Tao Feng, Jian-Hai Ding, Zheng-Hui Li, Yan Li, Qiong-Ying Fan, Ji-Kai Liu

**Affiliations:** 1 State Key Laboratory of Phytochemistry and Plant Resources in West China, Kunming Institute of Botany, Chinese Academy of Sciences, Kunming, 650201 China; 2 University of Chinese Academy of Sciences, Beijing, 100049 China; 3 Hebei Normal University, Hebei, 050024 China

**Keywords:** *Trichaptum biforme*, drimane sesquiterpenoids, cytotoxicity

## Abstract

Two new drimane sesquiterpenoids (**1** and **2**), as well as five known compounds (**3–7**), were isolated from the basidiomycete *Trichaptum biforme*. The structures of new compounds were elucidated by extensive spectroscopic methods, and the known compounds were identified by comparing their spectroscopic data with those reported in the literature. The cytotoxicities results against five human cancer cell lines of compounds **1** and **2** were negligible.

**Electronic Supplementary Material:**

Supplementary material is available for this article at 10.1007/s13659-013-0030-y and is accessible for authorized users.

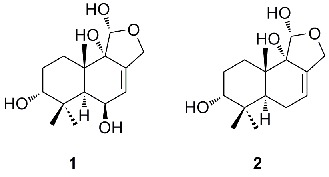

## Introduction

The *Trichaptum biforme* is an edible fungus belonging to the family Polyporaceae, which is mainly distributed in temperate regions and boreal forests zones throughout the world.[1] This fungus displays diverse pharmacological properties, including antimicrobial, antifungal,[2] decolorization of methylene blue,[3] degradation of pitch,[4] and delignification activities[5]. However, to date, the secondary metabolites of *T. biforme* have not been investigated further.[2] As a part of our search for diverse secondary metabolites from higher fungi,[6–11] we conducted chemical investigations on cultures of *T. biforme*, which led to the isolation of two new drimane sesquiterpenoids, 11,12-epoxy-3$\alpha$,6$\beta$,9$\alpha$,11$\alpha$-tetrahydroxydrimene (**1**) and 11,12-epoxy-3$\alpha$,9$\alpha$,11$\alpha$-trihydroxydrimene (**2**), together with four known analogues, danilol (**3**),[12] isodrimeninol (**4**),[13,14] 3$\beta$,11,12-trihydroxydrimene (**5**),[15,16] and 11,12-dihydroxydrimene (**6**),[16,17] as well as one chain-shaped sesquiterpenoid, 2,10-dodecadiene-1,6,7-triol (**7**).[18] Herein, we report the isolation and structural elucidation of **1** and **2**. All of these compounds were tested for their cytotoxicities against five human cancer cell lines.

| | R1 | R2 | R3 |
|---|---|---|---|
| **1** | $\alpha$-OH | OH | OH |
| **2** | $\alpha$-OH | H | OH |
| **3** | $\beta$-OH | H | H |
| **4** | H | H | H |

**5** R = OH
**6** R = H

**7**

## Results and Discussion

Compound **1** was obtained as white powder. Its molecular formula $C_{15}H_{24}O_5$ was deduced by the HRESIMS at *m/z* 307.1522 [M + Na]$^+$ (calcd 307.1521 for $C_{15}H_{24}O_5Na$), indicating four degrees of unsaturation. The IR spectrum showed absorption bands for hydroxy groups (3423 cm$^{-1}$) and C=C double bonds (1632 cm$^{-1}$). The $^{13}$C NMR spectrum revealed 15 carbon signals for three methyls, three methylenes (one oxygenated), five methines (one olefinic and three oxygenated), and four quaternary carbons (one olefinic and

*To whom correspondence should be addressed. E-mail: jkliu@mail.kib.ac.cn one oxygenated) (Table 1). Consideration of the above data led to the conclusion that **1** possessed three rings and four OH groups. The $^1$H-$^1$H COSY spectrum revealed the presence of the two partial structures as shown in Figure 1. The HMBC spectrum (Figure 1) of **1** showed correlations from both $\delta_H$ 1.36 (3H, s, Me-13) and 1.15 (3H, s, Me-14) to $\delta_C$ 78.7 (d, C-3), 39.3 (s, C-4), and 41.4 (d, C-5), and from $\delta_H$ 3.33 (1H, br. s, H-3) to C-5, indicated that C-3 and C-5 were attached to the quaternary carbon C-4. Furthermore, the HMBC correlations (Figure 1) from $\delta_H$ 2.43 (1H, m, H-1a) to $\delta_C$ 78.5 (s, C-9), 38.8 (s, C-10), and 19.4 (q, C-15), and from $\delta_H$ 2.30 (1H, d, H-5) to C-9, C-10, C-1, and C-15, revealed that C-1 and C-9 were attached to the quaternary carbon C-10 and the connectivity from C-5 to C-10. In addition, the HMBC correlations (Figure 1) from $\delta_H$ 4.19 (1H, dt, H-12b) to C-7, C-11, and C-9 revealed the connectivity of C-12 to C-8 and C-11 to C-12 via an oxygen atom, which formed a five-membered ring. The data described above gave a gross structure of **1** belonging to a drimane sesquiterpenoid, which was related to that of danilol (**3**),[8] except for two more hydroxy groups at C-6 and C-9 in **1**. The relative configuration of **1** was deduced from the ROESY spectrum (Figure 1). Biogenetically, the methyl group of Me-15 was $\beta$-oriented, whilst H-5 was $\alpha$-oriented.[8] Therefore, the key ROESY correlation between Me-15 and H-11 allowed H-11 to be $\beta$-oriented, while the ROESY correlation of H-5/H-6 indicated that H-6 was $\beta$-oriented, Moreover, a 3D structure model of the ROESY correlation of Me-15/H-11 was determined and it was revealed that the OH-9 could only be $\alpha$-oriented (Figure 1). The broad peak of H-3 indicated that the OH-3 was $\alpha$-oriented. Therefore, compound **1** was elucidated as 11,12-epoxy-3$\alpha$,6$\beta$,9,11$\alpha$-tetrahydroxydrimene.

Compound **2** was deduced as the molecular formula $C_{15}H_{24}O_4$ by the HRESIMS at *m/z* 291.1567 [M + Na]$^+$ (calcd 291.1572 for $C_{15}H_{24}O_4Na$). With the assistance of 2D ($^1$H-$^1$H COSY, HSQC, HMBC) NMR data, compound **2** was shown to possess the same tricyclic carbon skeleton as that of **1**. Furthermore, a comparison of the $^{13}$C NMR data with those of **1** (Tables 1) showed that they were similar, except for the absence of a hydroxy group at C-6 in **2**. This assignment was

**Figure 1.** Key 2D NMR correlations of **1**

confirmed by $^1$H-$^1$H COSY correlations of $\delta_H$ 2.04 (1H, m, H-6a) and 1.99 (1H, m, H-6b) with $\delta_H$ 5.68 (1H, m, H-7) and HMBC correlations from H-6 to $\delta_C$ 122.9 (d, C-7) and 139.0 (s, C-8). The broad peak of H-3 ($\delta_H$ 3.38, 1H, br. s) indicated that OH-3 was $\alpha$-oriented. The ROESY correlations of Me-15/H-11 indicated that the relative configuration at C-9 and C-11 in **2** was the same as that of **1**. Thus, compound **2** was elucidated as 11,12-epoxy-3$\alpha$,9,11$\alpha$-trihydroxydrimene.

Compounds **1** and **2** were evaluated for their cytotoxicities against five human cancer cell lines namely: SK-BR-3 breast, SMMC-7721 hepatocellular carcinoma, HL-60 myeloid leukemia, PANC-1 pancreatic cancer, and A-549 lung cancer cell lines, using the MTT method as reported previously.[19] Unfortunately, nither compound **1** or **2** displayed any cytotocicity activity againist these cancer cell lines ($IC_{50}$ > 40 $\mu$M).

## Experimental Section

**General Experimental Procedures.** Optical rotations were measured on a Horiba SEPA-300 polarimeter. IR spectra were obtained on a Bruker Tensor 27 spectrometer with KBr pellets. 1D and 2D NMR experiments were performed on a Bruker AM-400, DRX-500 or AVANCE III-600 spectrometer with TMS as the internal standard. Chemical shifts ($\delta$) were expressed in ppm with reference to the solvent signals. Mass spectra (MS) were recorded on a VG Auto Spec-3000 or an

**Table 1.** $^1$**H (400 MHz) and** $^{13}$**C (100 MHz) NMR data of 1 and 2 in methanol-*d*$_4$**

| | 1 | | 2 | |
|---|---|---|---|---|
| no. | $\delta_H$ | $\delta_C$ | $\delta_H$ | $\delta_C$ |
| 1 | 2.43, m, H$_a$; 1.00, m, H$_b$ | 25.9, t | 2.31, m, H$_a$; 1.00, m, H$_b$ | 25.1, t |
| 2 | 2.04, m, H$_a$; 1.59, m, H$_b$ | 25.2, t | 1.92, m, H$_a$; 1.59, m, H$_b$ | 25.7, t |
| 3 | 3.33, br. s | 78.7, d | 3.38, br. s | 76.7, d |
| 4 | | 39.3, s | | 38.9, s |
| 5 | 2.30, d (5.0) | 41.4, d | 2.31, overlap | 37.6, d |
| 6 | 4.40, m | 66.4, d | 2.04, m, H$_a$,; 1.99, m, H$_b$ | 24.8, t |
| 7 | 5.67, m | 124.8, d | 5.68, m | 122.9, d |
| 8 | | 140.1, s | | 139.0, s |
| 9 | | 78.5, s | | 78.5, s |
| 10 | | 38.8, s | | 38.2, s |
| 11 | 5.34, s | 99.3, d | 5.26, s | 99.1, d |
| 12 | 4.56, dt (12.4, 2.4, H$_a$); 4.19, dt (12.4, 1.4, H$_b$) | 67.9, t | 4.50, ddd (11.7, 5.3, 3.0, H$_a$); 4.08, ddd (11.7, 3.5, 1.9, H$_b$) | 68.2, t |
| 13 | 1.36, s | 25.6, q | 0.97, s | 22.9, q |
| 14 | 1.15, s | 28.4, q | 0.96, s | 29.1, q |
| 15 | 1.18, s | 19.4, q | 0.90, s | 16.5, q |

APIQSTAR time-of-flight spectrometer. Column chromatography (CC) was performed using a silica gel (200–300 mesh; Qingdao Marine Chemical Co., Ltd., China), and Sephadex LH-20 (Amersham Biosciences, Sweden). Fractions were monitored for qualitative analysis using TLC ($GF_{254}$, Qingdao Marine Chemical Co., Ltd., China), and spots were visualized by spraying with 10% $H_2SO_4$ in ethanol.

**Fungal Material and Cultivation Condition.** *T. biforme* was isolated from a tissue culture of its fruiting bodies, which was originally collected from Changbai Mountain in Jilin Province, China in 2009. The specimen was authenticated by Prof. Tuli Guer from Jilin Agricultural University. A voucher specimen was deposited in the Herbarium of Kunming Institute of Botany, Chinese Academy of Sciences. The culture medium consisted of glucose 5%, peptone 0.15%, yeast powder 0.5%, $KH_2PO_4$ 0.05% and $MgSO_4$ 0.05%. Fermentation was conducted using a shaker at 24 °C and 150 rpm for 26 days.

**Extraction and Isolation.** The culture broth of *T. biforme* (20 L) was filtered, and the filtrate was extracted three times with EtOAc. The organic layer was concentrated under reduced pressure to give an oily residue (3.9 g) that was subjected to column chromatography (CC) over silica gel (200–300 mesh) eluting with $CHCl_3$/MeOH (from 100:0 to 0:100) to derive fractions A–G. Fraction A (130 mg) was isolated by repeated CC on silica gel (petroleum ether-$Me_2CO$) to give compounds **4** (2.3 mg), **5** (1.2 mg) and **6** (1.9 mg). Fraction C (88 mg) was subjected to Sephadex LH-20 CC ($Me_2CO$) and purified by repeated CC on silica gel (petroleum ether-$Me_2CO$) to derive compounds **3** (2.5 mg) and **7** (3.7 mg). Fraction E (600 mg) was subjected to Sephadex LH-20 CC ($CHCl_3$-MeOH, 1:1) and purified by column chromatography on silica gel eluted with petroleum ether-acetone (3:1) to derive compound **2** (45.8 mg). Fraction G (200 mg) was isolated first by CC on silica gel eluted with petroleum ether-EtOAc (1:4) and purified by Sephadex LH-20 ($CHCl_3$-MeOH, 1:1) to afford compound **1** (21.3 mg).

**11,12-Epoxy-3$\alpha$,6$\beta$,9$\alpha$,11$\alpha$-tetrahydroxydrimene (1):** colorless oil; $[\alpha]_D^{25}$ – 163.0 (*c* 0.36, $CHCl_3$); $^1H$ NMR (methanol-$d_4$, 400 MHz) and $^{13}C$ NMR (methanol-$d_4$, 100 MHz) data, see Table 1; IR (KBr) $\nu_{max}$: 3423, 2924, 2872, 1632, 1385, 1151, 1047, 1026, 911 $cm^{-1}$; ESIMS (pos.) *m/z* 307 ($[M + Na]^+$, 100); ESIHRMS *m/z* 307.1522 (calcd for $C_{15}H_{24}O_5Na$, 307.1521).

**11,12-Epoxy-3$\alpha$,9$\alpha$,11$\alpha$-trihydroxydrimene (2):** colorless oil; $[\alpha]_D^{26}$ – 95.9 (*c* 0.65, $CHCl_3$); $^1H$ NMR (methanol-$d_4$, 400 MHz) and $^{13}C$ NMR (methanol-$d_4$, 100 MHz) data, see Table 1; IR (KBr) $\nu_{max}$ 3483, 3361, 3280, 2971, 2925, 2878, 1630, 1434, 1389, 1125, 1101, 1056, 1041, 999, 907 $cm^{-1}$; ESIMS (pos.) *m/z* 291 ($[M + Na]^+$, 100); ESIHRMS *m/z* 291.1567 (calcd for $C_{15}H_{24}O_4Na$ 291.1572).

**Cytotoxicity Assay.** Five human cancer cell lines: SK-BR-3 breast, SMMC-7721 hepatocellular carcinoma, HL-60 myeloid leukemia, PANC-1 pancreatic cancer and A-549 lung cancer. All the cells were cultured in RPMI-1640 or DMEM medium (Hyclone, USA), supplemented with 10% fetal bovine serum (Hyclone, USA) in 5% $CO_2$ at 37 °C. The cytotoxicity assay was performed according to the MTT (3-(4,5-dimethylthiazol-2-yl)-2,5-diphenyl tetrazolium bromide) method in 96-well microplates. Briefly, 100 $\mu$L adherent cells were seeded into each well of 96-well cell culture plates and allowed to adhere for 12 h before isolated compound addition. The suspended cells were seeded just before the isolated compounds were added with an initial density of $1 \times 10^5$ cells/mL. Each tumor cell line was exposed to the test compound at concentrations of 0.0625, 0.32, 1.6, 8, and 40 $\mu$M in triplicates for 48 h, with cisplatin (sigma, USA) as a positive control. After the compound treatment, cell viability were detected and cell growth curve was graphed.

## Electronic Supplementary Material

Supplementary material is available in the online version of this article at http://dx.doi.org/10.1007/s13659-013-0030-y and is accessible for authorized users.

## Acknowledgments

This project was supported by the National Basic Research Program of China (973 Program, 2009CB522300), the National Natural Sciences Foundation of China (U1132607), and Youth Innovation Promotion Association of Chinese Academy of Sciences.

**Open Access** This article is distributed under the terms of the Creative Commons Attribution License which permits any use, distribution, and reproduction in any medium, provided the original author(s) and source are credited.

## Electronic supplementary material

Supplementary material, approximately 1.01 MB.
