# Supplementary material for: Two new drimane sesquiterpenoids from cultures of the basidiomycete Trichaptum biforme
Source: Nat Prod Bioprospect. 2013 Jul 11;3(4):154–7. doi: 10.1007/s13659-013-0030-y (PMC4131575; doi:10.1007/s13659-013-0030-y)
Supplement: Supplementary file 1 — Supplementary material, approximately 1.01 MB. [file 13659_2013_30_MOESM1_ESM.pdf]

Electronic Supplementary Material

Two new drimane sesquiterpenoids from cultures of the basidiomycete *Trichaptum biforme*

Xiao-Yan YANG,[a,b] Tao FENG,[a] Jian-Hai DING,[a,b] Zheng-Hui LI,[a] Yan LI,[a] Qiong-Ying FAN,[a,c] and Ji-Kai LIU[a,]*

[a]State Key Laboratory of Phytochemistry and Plant Resources in West China, Kunming Institute of Botany, Chinese Academy of Sciences, Kunming 650201, China

[b]University of Chinese Academy of Sciences, Beijing 100049, China

[c]Hebei Normal University, Hebei 050024, China

Received 9 March 2013; Accepted 16 June 2013

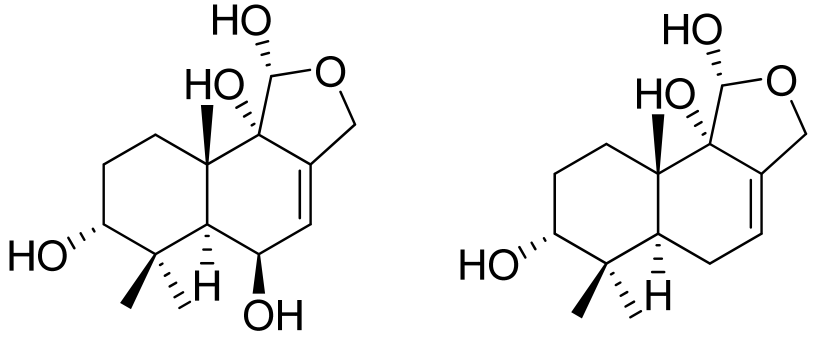

1 **2**

Structures of compounds **1** and **2**

*To whom correspondence should be addressed. E-mail: jkliu@mail.kib.ac.cn

Contents

1D and 2D NMR and MS of compound **1**.......................................S3-S6
1D and 2D NMR and MS of compound **2**.......................................S7-S10

^{1}H NMR(400 MHz) spectrum of compound **1** in methanol-d_4.

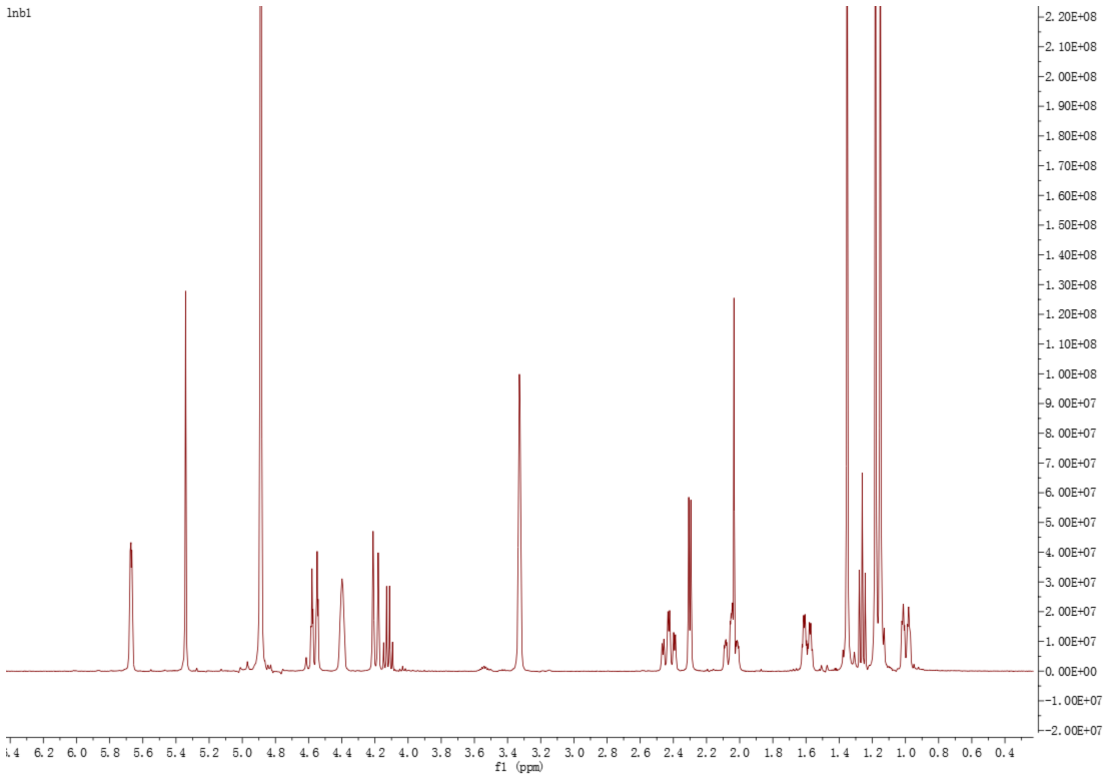

^{13}C NMR(100 MHz) spectrum of compound **1** in methanol-d_4.

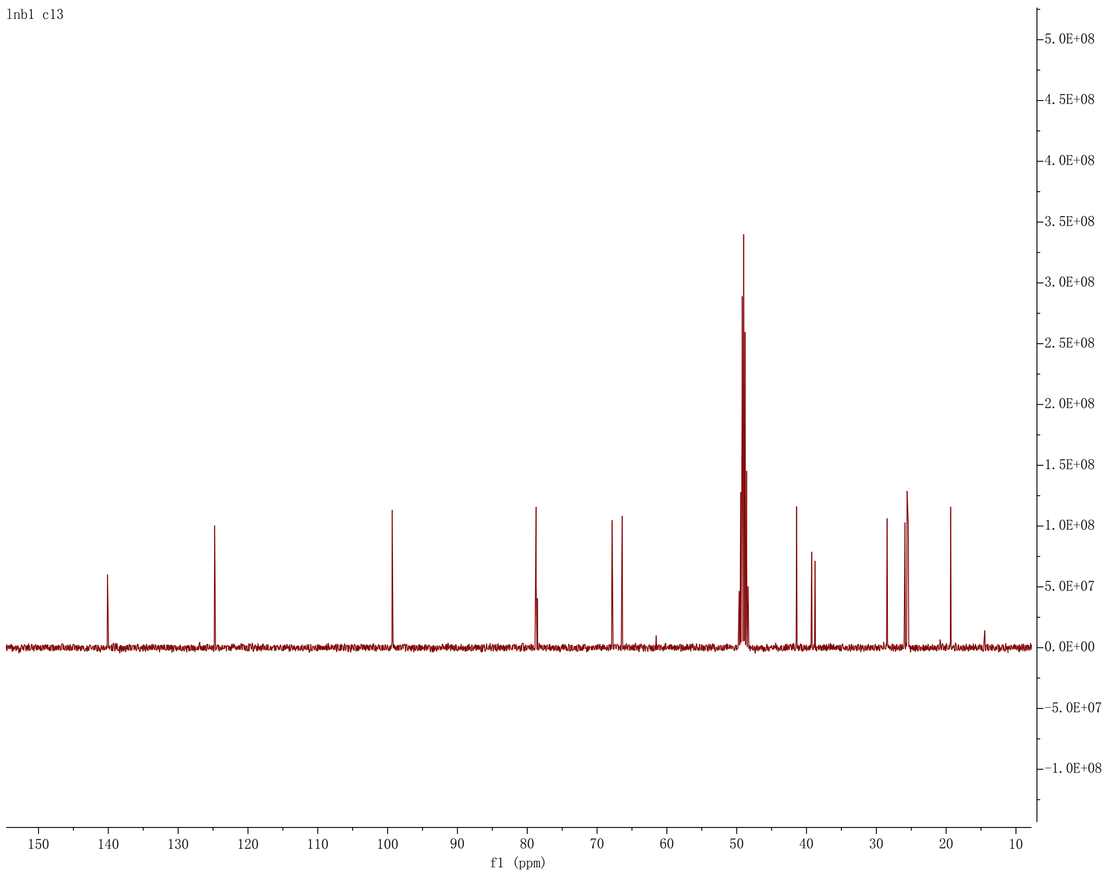

HSQC (500 MHz) spectrum of compound **1** in methanol-d_4.

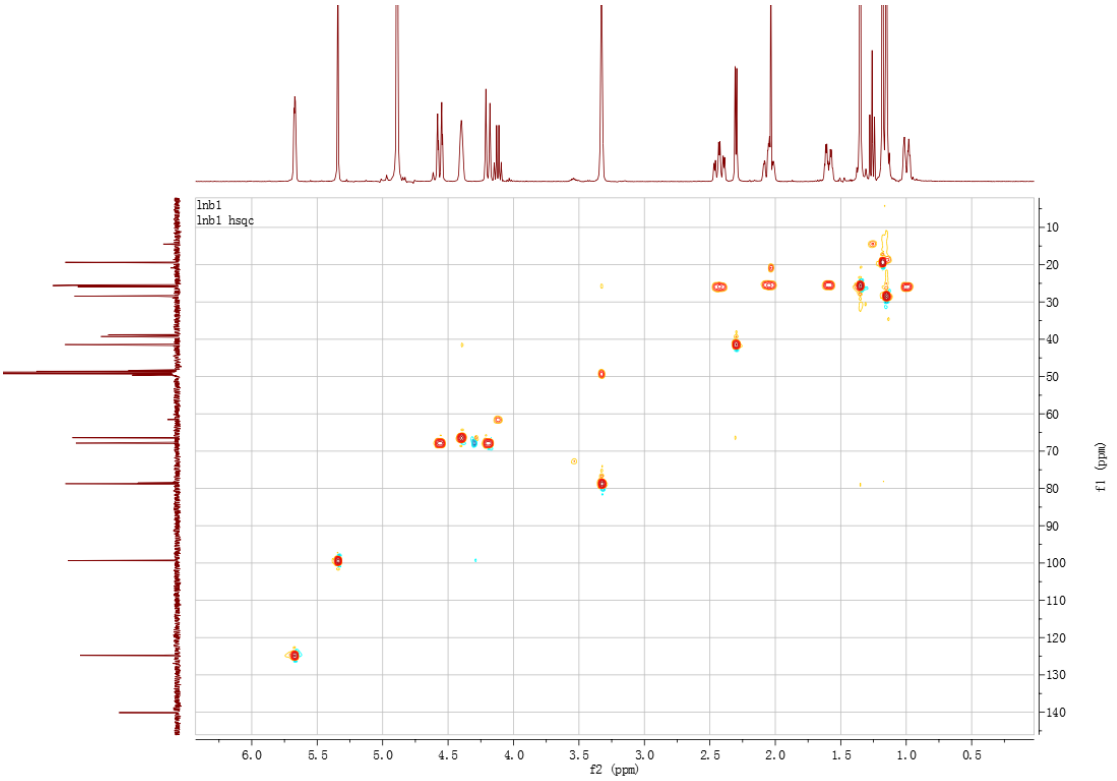

HMBC (500 MHz) spectrum of compound **1** in methanol-d_4.

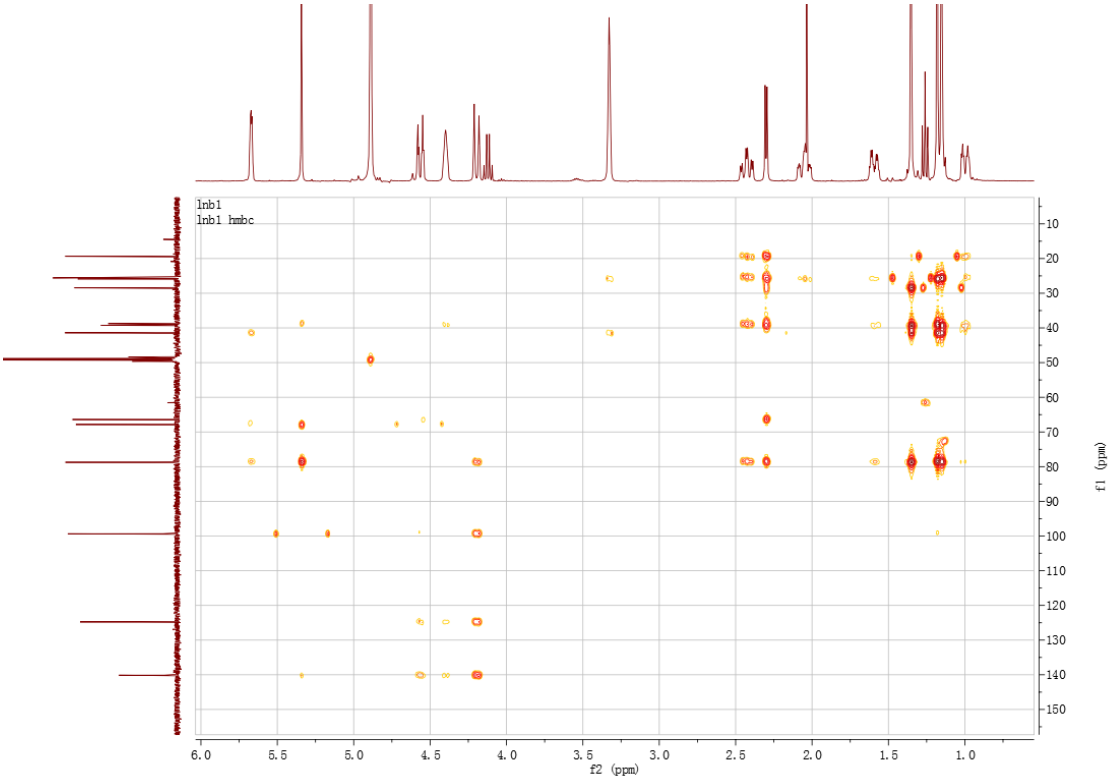

^{1}H-^{1}H COSY (500 MHz) spectrum of compound **1** in methanol-d_4.

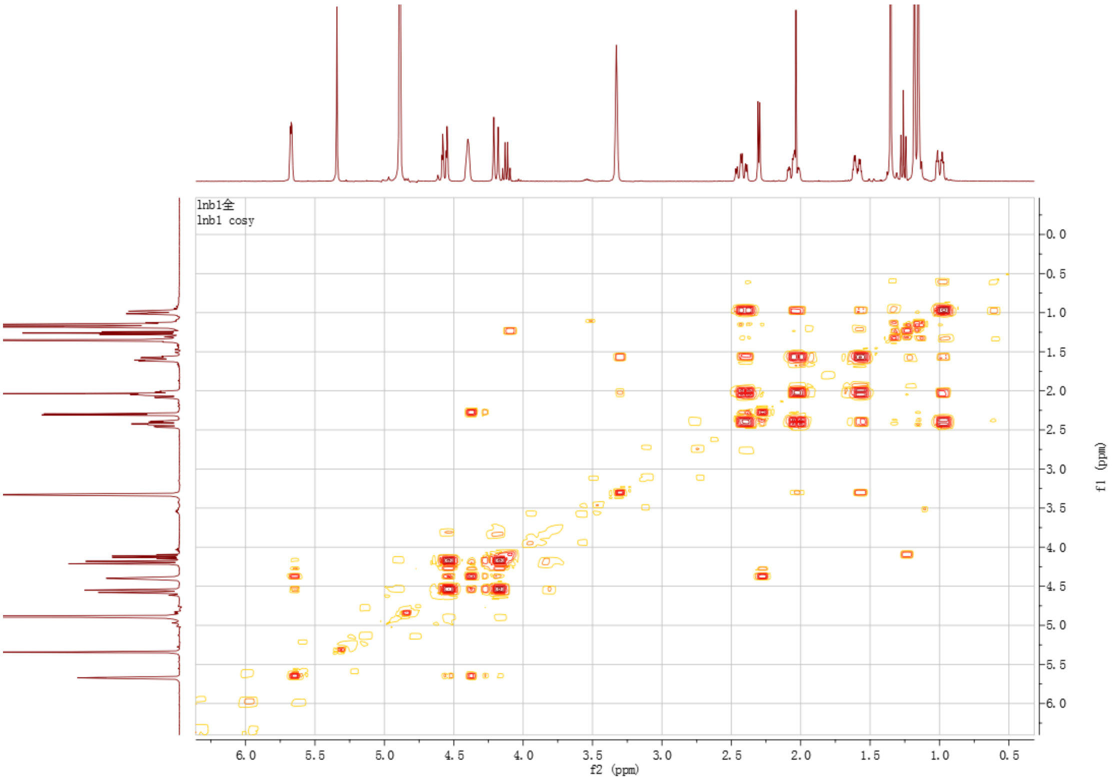

ROESY(500 MHz) spectrum of compound **1** in methanol-d_4.

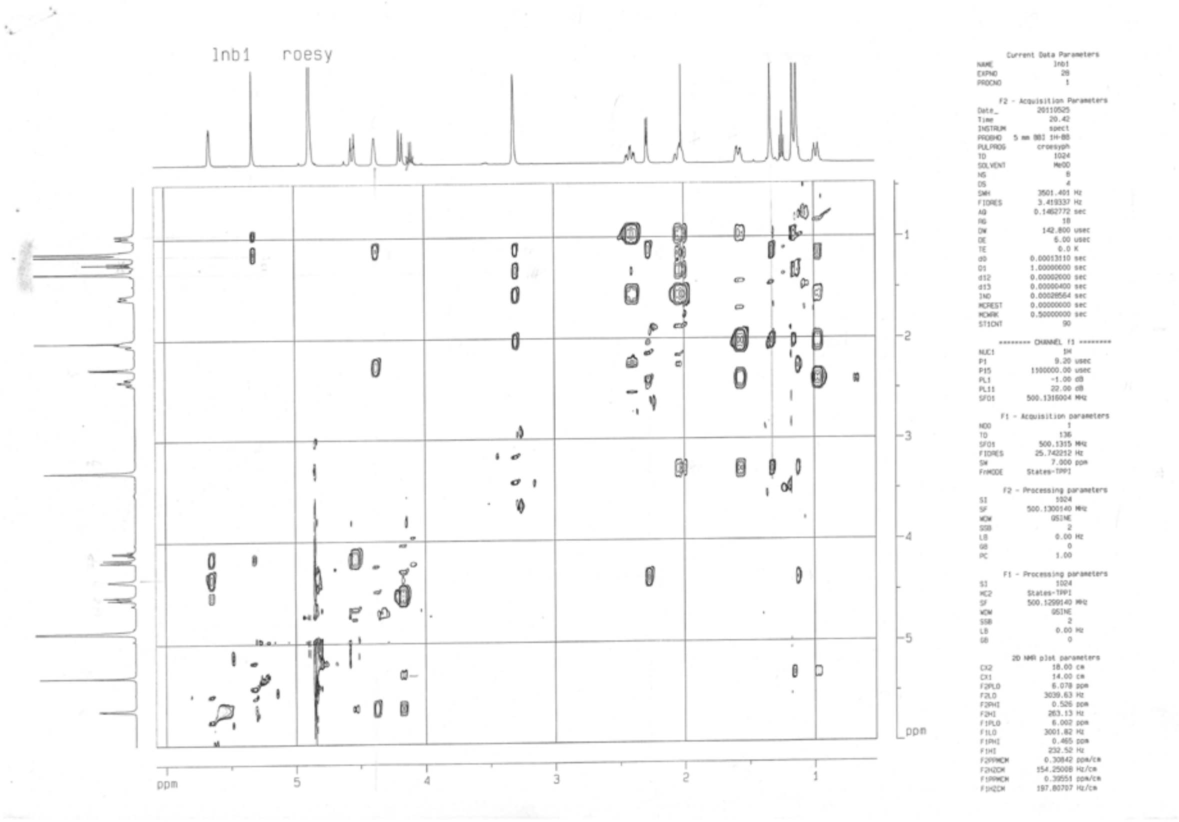

HRESIMS of compound **1**.

Acq. Date: Friday, September 09, 2011 Acq. Time: 15:21

Sample Name: 110910ESIA lnb1

Elemental composition calculator

Target m/z: +307.1522 amu
Tolerance: +10.0000 ppm
Result type: Elemental
Max num of results: 1000
Min DBE: -10.0000 Max DBE: +60.0000
Electron state: OddAndEven
Num of charges: 0
Add water: N/A
Add proton: N/A
File Name: 110910ESIA lnb1.wiff

	Elements	Min Number	Max Number
1	Br	0	0
2	C	0	200
3	Cl	0	0
4	F	0	0
5	H	0	400
6	I	0	0
7	K	0	0
8	N	0	0
9	Na	1	1
10	O	2	5

^{1}H NMR (400 MHz) spectrum of compound **2** in methanol-d_4.

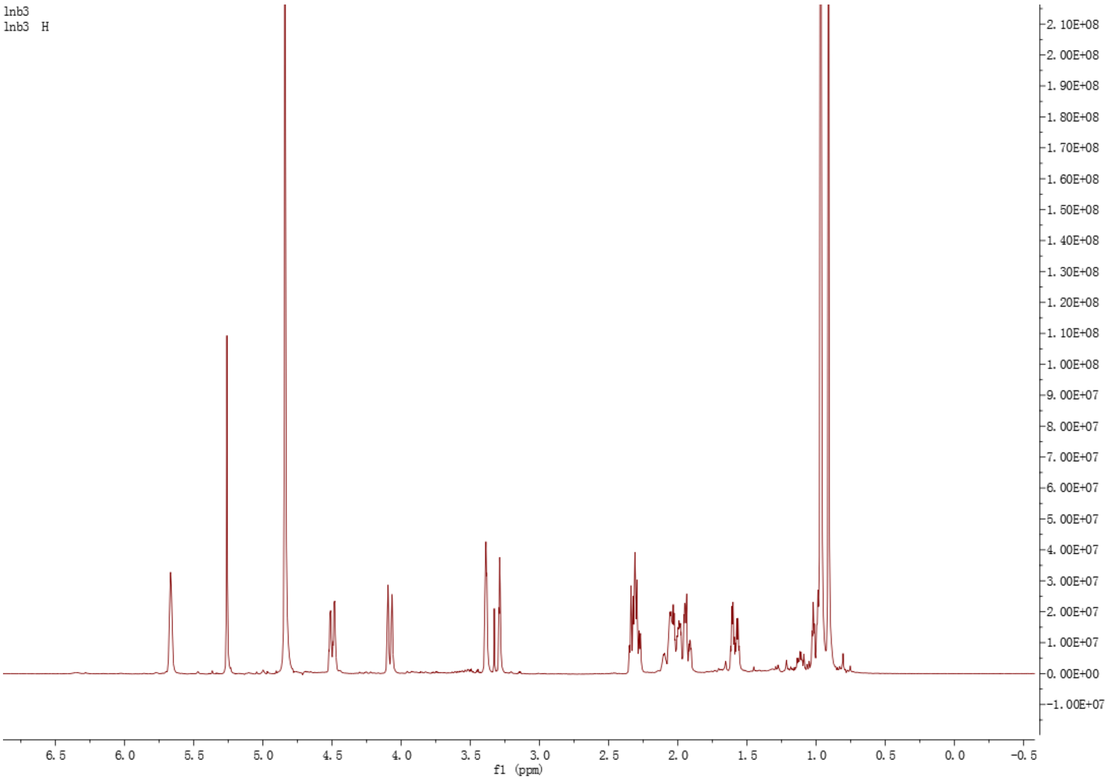

^{13}C NMR (100 MHz) spectrum of compound **2** in methanol-d_4.

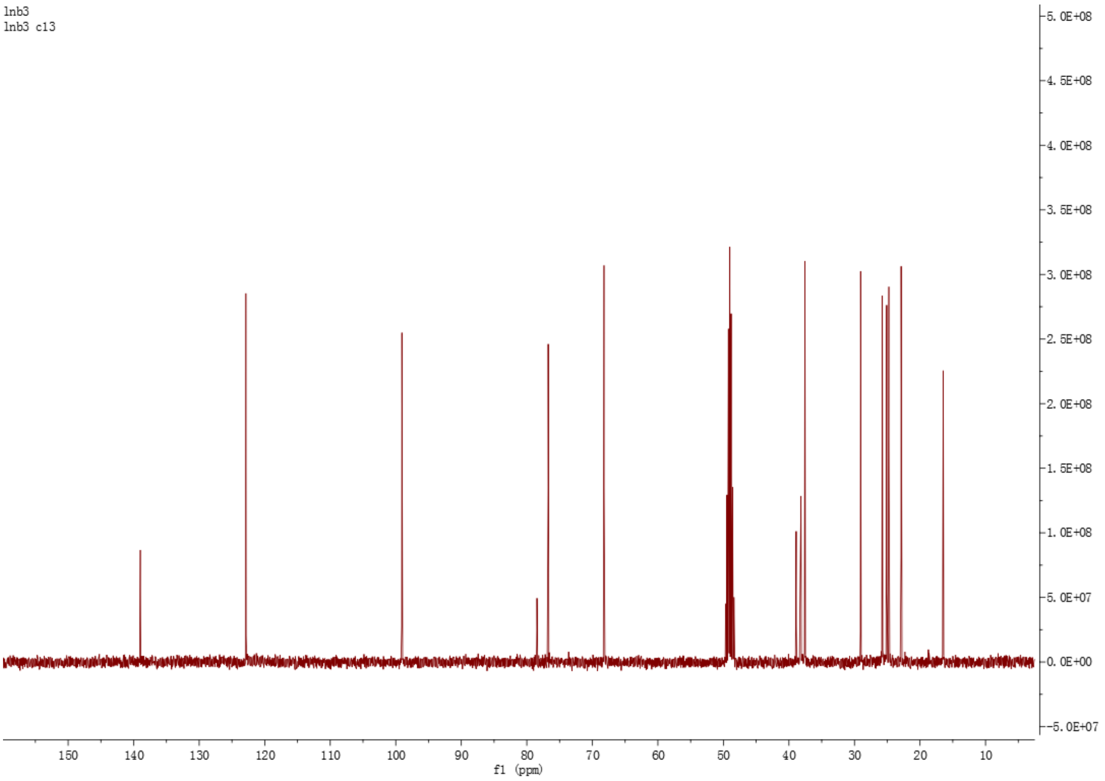

HSQC (500 MHz) spectrum of compound **2** in methanol-d_4.

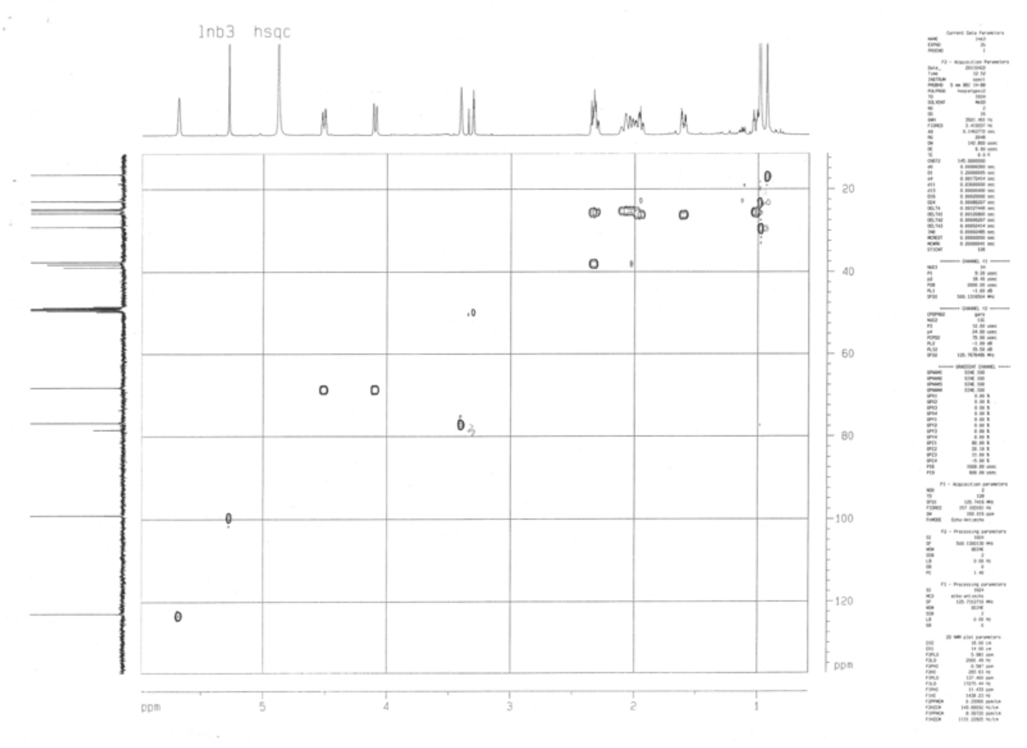

HMBC (500 MHz) spectrum of compound **2** in methanol-d_4.

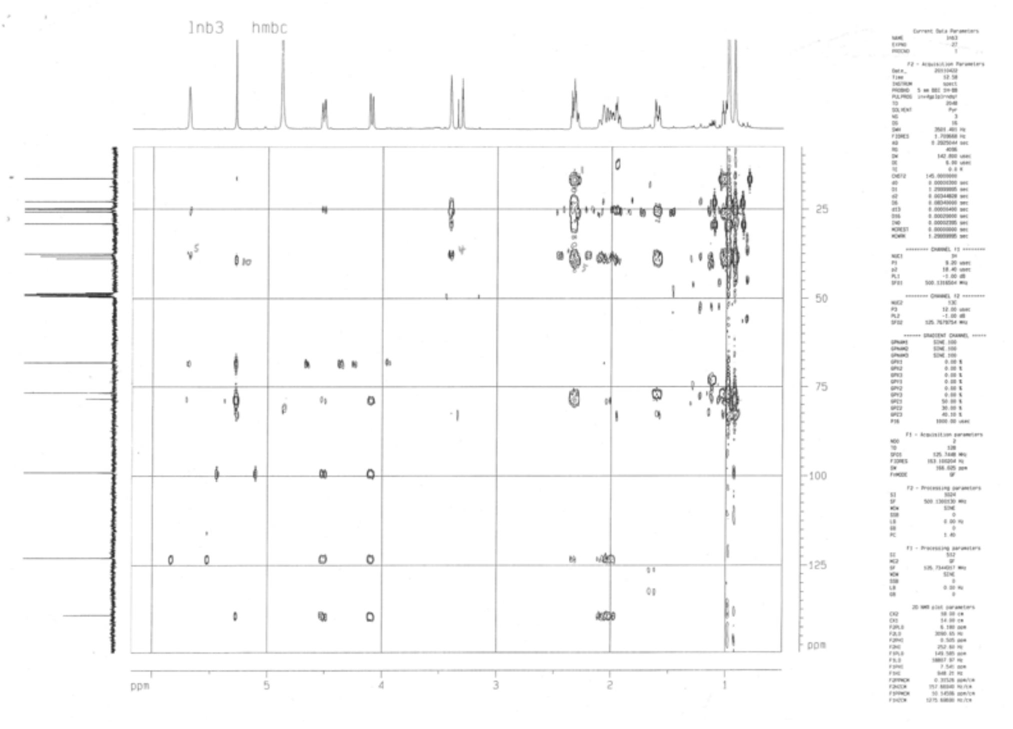

^{1}H-^{1}H COSY (500 MHz) spectrum of compound **1** in methanol-d_4.

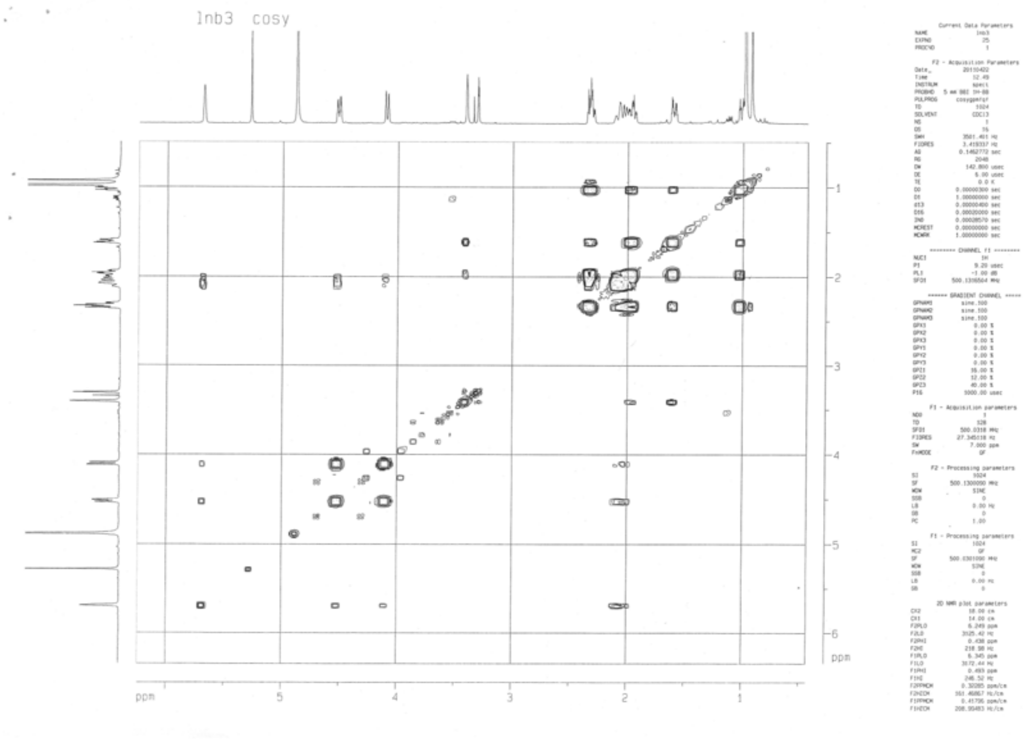

ROESY (500 MHz) spectrum of compound **2** in methanol-d_4.

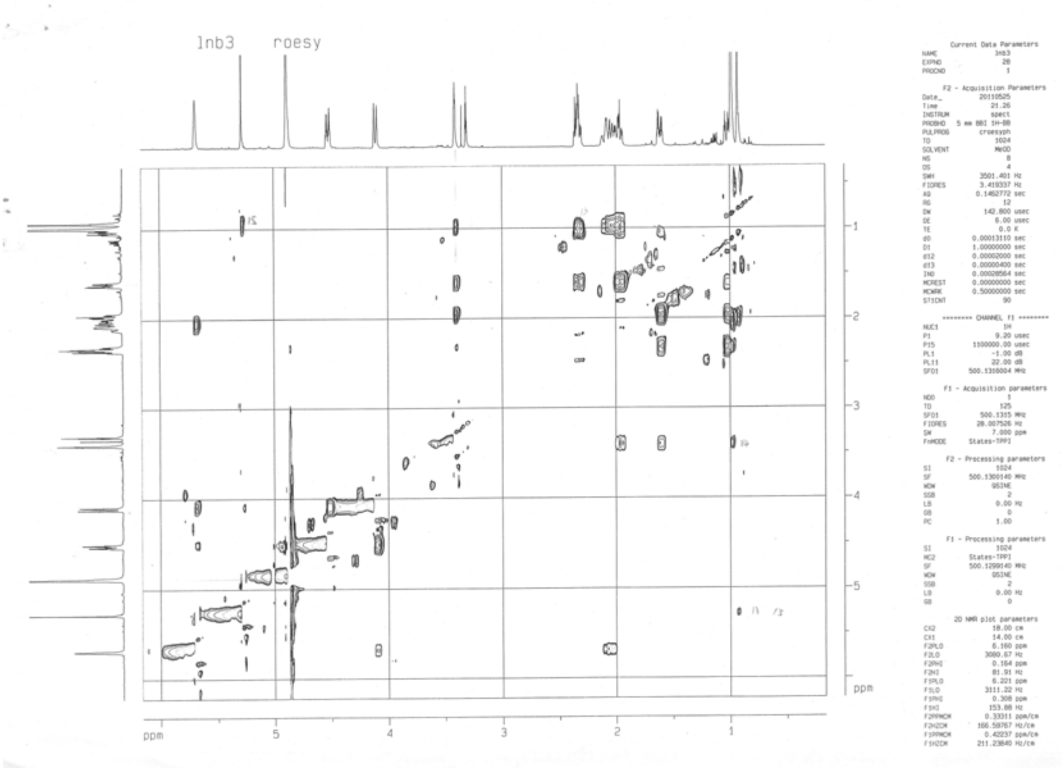

HRESIMS of compound **2**.

Acq. Date: Friday, September 09, 2011 Acq. Time: 15:17

Sample Name: 110910ESIA lnb3

Elemental composition calculator

Target m/z: +291.1567 amu
Tolerance: +10.0000 ppm
Result type: Elemental
Max num of results: 1000
Min DBE: -10.0000 Max DBE: +60.0000
Electron state: OddAndEven
Num of charges: 0
Add water: N/A
Add proton: N/A
File Name: 110910ESIA lnb3.wiff

	Elements	Min Number	Max Number
1	Br	0	0
2	C	0	200
3	Cl	0	0
4	F	0	0
5	H	0	400
6	I	0	0
7	K	0	0
8	N	0	0
9	Na	1	1
10	O	0	4
